# Orientation selective stimulation with tetrahedral electrodes of the rat infralimbic cortex to indirectly target the amygdala

**DOI:** 10.3389/fnins.2023.1147547

**Published:** 2023-05-05

**Authors:** Irina Gureviciene, Hanne Laakso, Omar Narvaez, Ekaterina Paasonen, Lauri Lehto, Kestutis Gurevicius, Silvia Mangia, Shalom Michaeli, Olli Gröhn, Alejandra Sierra, Heikki Tanila

**Affiliations:** ^1^A. I. Virtanen Institute for Molecular Sciences, University of Eastern Finland, Kuopio, Finland; ^2^Center for Magnetic Resonance Research, University of Minnesota, Minneapolis, MN, United States

**Keywords:** deep brain stimulation, medial frontal cortex, depression, evoked response, MRI, functional connectivity

## Abstract

**Introduction:**

Deep brain stimulation (DBS) is a rapidly developing therapeutic intervention with constantly expanding neurological and psychiatric indications. A major challenge for the approach is the precise targeting and limitation of the effect on the desired neural pathways. We have introduced a new approach, orientation selective stimulation (OSS) that allows free rotation of the induced electric field on a plane when using a probe with three parallel electrodes forming an equilateral triangle at the tip. Here, we expand the technique by introducing a tetrahedral stimulation probe that enables adjustment of the primary electric field direction freely at any angle in a 3D space around the stimulating probe. OSS in 3D will enable better targeting of the electric field according to the local brain anatomy. We tested its utility in a rat model of DBS for treatment-resistant depression. The stimulation directed to the subgenual anterior cingulate cortex (sgACC) has yielded dramatic improvement in individual patients suffering from therapy resistant depression, but no consistent benefit in larger series. This failure has been ascribed to the challenging anatomy of sgACC with several crossing neural tracts and individual differences in the local anatomy.

**Methods:**

We stimulated infralimbic cortex (IL), the rat analog of sgACC, and recorded local electrical responses in amygdala (AMG) that is monosynaptically connected to IL and plays a central role in emotional states. We further traced AMG–IL connections using a viral vector and tractography using diffusion magnetic resonance imaging (MRI). Finally, we mimicked the clinical situation by delivering sustained 130 Hz stimulation at IL at the most effective field orientation and followed changes in resting-state functional connectivity with IL using functional MRI. To help interpretation of responses in functional connectivity, we stimulated only the left IL, which we did not expect to evoke measurable changes in the rat behavior.

**Results:**

The AMG evoked responses depended systematically on the IL stimulation field orientation and yielded the maximum response in near vertical field orientation in accordance with tractography. Sustained 130 Hz stimulation at a field orientation yielding the strongest AMG evoked responses increased functional connectivity between IL and AMG on the stimulation side.

**Conclusion:**

These findings suggest that OSS in 3D provides a new approach to optimize the DBS for every individual patient with a single stimulation probe implantation.

## 1. Introduction

Deep brain stimulation (DBS) has become an established clinical treatment for severe Parkinson’s disease, essential tremor and dystonia, and is under experimental phase for several other neurological and psychiatric conditions. Through trial and error, high-frequency stimulation around 150 Hz has become the standard stimulation pattern in most applications with the idea of suppressing a hyperactive nucleus, such as the subthalamic nucleus in the case of Parkinson’s disease (McIntyre et al., 2004). There are several theories how high-frequency stimulation works but a current understanding is that suppression of neuronal somata in the target nucleus is accompanied by activation of the axons at the stimulation frequency (McIntyre et al., 2004). This explains the major challenge of DBS, undesired motor effect by stimulation of nearby neuronal tracts. To avoid these, targeting of the electric field has become the focus of the DBS electrode design.

**Figure 1:**
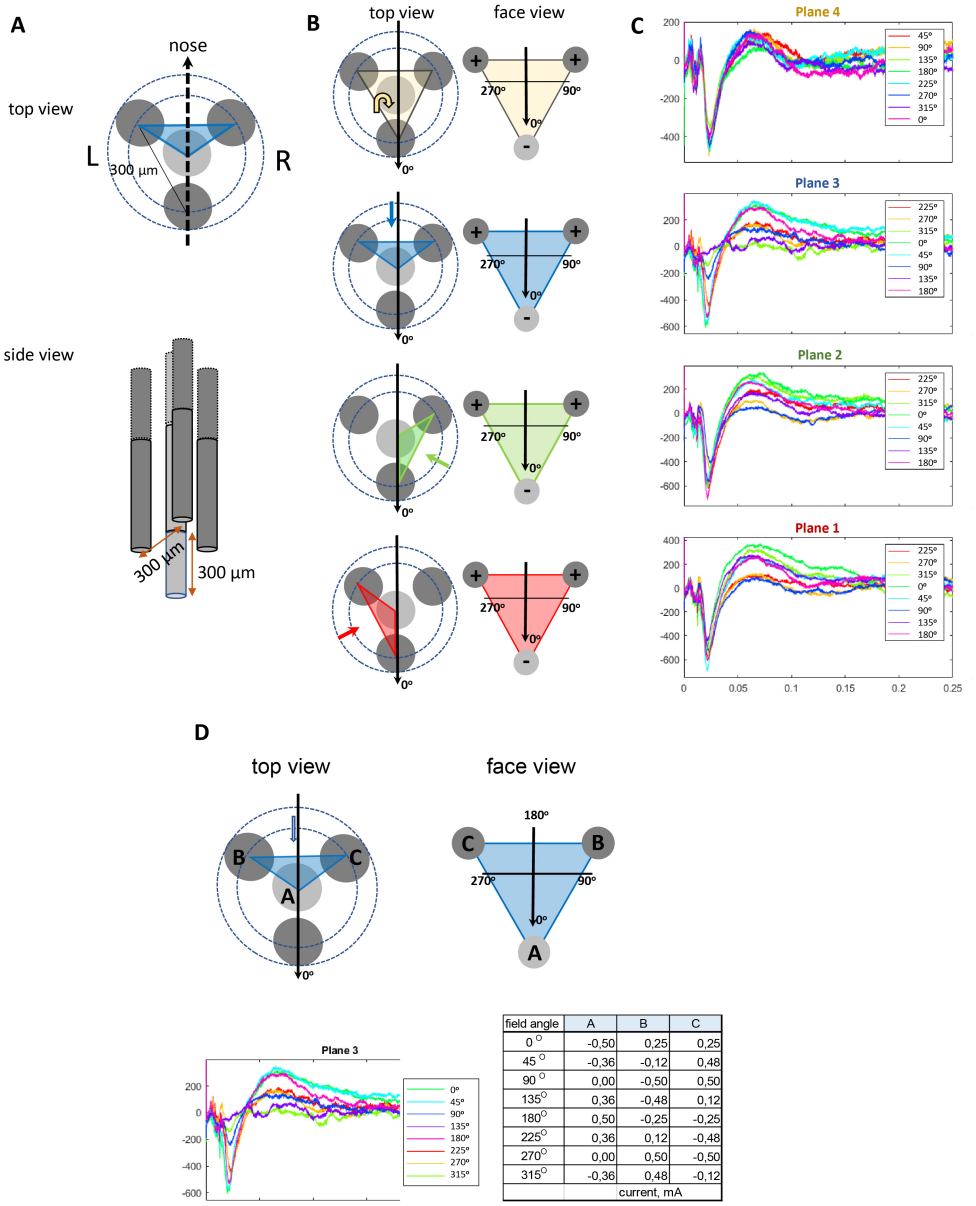
Schematic illustration of the orientation selective stimulation with a tetrahedral probe. **(A)** The probe consisted of four insulated wires, three of which were positioned 300 μm apart so that their tips formed an equilateral triangle. The fourth wire was correspondingly 300 μm longer to complete a tetrahedron. **(B)** The stimulation angles in the text are given separately for each plane (face of the tetrahedron, indicated by color codes, the direction of view indicated by the colored arrow) in local coordinates. The field direction 0^o^ (indicated by the black arrow) was set to point ventrally on Planes 1–3 and toward the tail on Plane 4. The charge on the electrodes shows the case of this particular field direction. **(C)** Examples of evoked local AMG responses to IL stimulation at 8 different field orientations in each of the 4 planes in one rat. **(D)** An example case of one side face (Plane 3) of the tetrahedron. The edges of the tetrahedron **(A–C)** correspond to the three active electrode tips. The table gives the actual current intensities (mA) of the three electrodes to obtain the given field angles. The corresponding AMG responses for each field angle are separated by the color code.

Conventional cylindrical DBS probes with a few axial electrode rings allow direction of the electric field only parallel or perpendicular to the probe shank, whereas the new generation directional electrodes with segmented rows of electrodes also allow bipolar stimulation diagonally between the electrodes (Slopsema et al., 2018; Janson et al., 2020). We have recently introduced an alternate approach, called orientation selective stimulation (OSS) that allows adjustment of the primary electric field direction freely at any angle on a plane around a three-electrode stimulating probe (Lehto et al., 2018). The idea is the same as in the three-phase electric power, such that the relative current amplitudes of the three channels are chosen based on sinusoidal functions with phase offsets of 120°. Thus, the sum of the instantaneous currents in each electrode is zero and the current in each electrode is equal in magnitude to the sum of the currents in the other two, but with the opposite sign. During the full sinusoidal cycle, the electrical field rotates 360^o^. A comparison of local fMRI responses in the somatosensory cortex to thalamic directional current steering stimulation vs. OSS in a swine model was recently published (Slopsema et al., 2021). To allow adjustment of the primary electric field in full three dimensions, we developed a tetrahedral four electrode probe. Here the three-phase principle can be applied in each of the four triangular faces of the tetrahedron, resulting in 3D-OSS (Figure 1).

To test the *in vivo* applicability of 3D-OSS, we chose to model DBS for the treatment-resistant depression, which has proved to be technically particularly challenging. Major depressive disorder is the leading cause of long-term disability and diminishes quality of life (Wittchen et al., 2010). Even though antidepressant medication alone or combined with psychotherapy is usually effective, about 30% of patients are resistant to medical therapy (Zhdanava et al., 2021). DBS has been offered as treatment to the most difficult cases, with severe and pervasive symptoms, co-morbidities, and exposure to many consecutive conventional treatments (including electroconvulsive therapy). Among the chosen target areas, DBS at subgenual anterior cingulate cortex (sgACC) has been reported to yield striking and sustained beneficial responses reported in individual patients (Mayberg et al., 2005). However, a recent double-blind sham-controlled clinical study comprising 90 patients could not find a statistically significant beneficial effect of the treatment due to large individual variability in the treatment response, which also was assessed with relatively crude measures (Holtzheimer et al., 2017). One likely reason to heterogeneous responses to sgACC DBS is the challenging anatomy of the target region. The typical stimulation site lies at the crossroad of four major axonal pathways: forceps minor, cingulum bundle, uncinate fasciculus, and subcortical connections between the frontal pole and the thalamus or ventral striatum (Howell et al., 2019). Recent studies have identified pathways with favorable clinical responses (Howell et al., 2019), but the high individual variability in the local anatomy makes it very challenging task to insert the stimulation probe in the optimal position during the stereotactic surgery. Due to the challenging anatomy and the promises of clinical significance, we chose the infralimbic cortex (IL) on the medial frontal cortex, the rat homolog of sgACC (Gabbott et al., 2003; Uylings et al., 2003), as the brain site to test the utility of 3D-OSS.

Human tractography studies have identified the sgACC to have strongest connections with the amygdala (AMG), nucleus accumbens, hypothalamus, and orbitofrontal cortex (Johansen-Berg et al., 2008). Corresponding connections of the rat IL to AMG, nucleus accumbens and hypothalamus have been recently verified with combined viral vector tracing and cFos studies (Wood et al., 2019). Furthermore, DBS targeted to IL at a corresponding frequency of 130 Hz that has been used in human studies has been shown to have antidepressant-like effect in rats (Hamani et al., 2010; Jiménez-Sánchez et al., 2016). As AMG has strong reciprocal connections with IL, is located far beyond the zone of passive currents spread, and plays a key role in emotional reactions, we chose it as the site to assess the impact of modulating the DBS current field orientation in IL.

**Figure 2:**
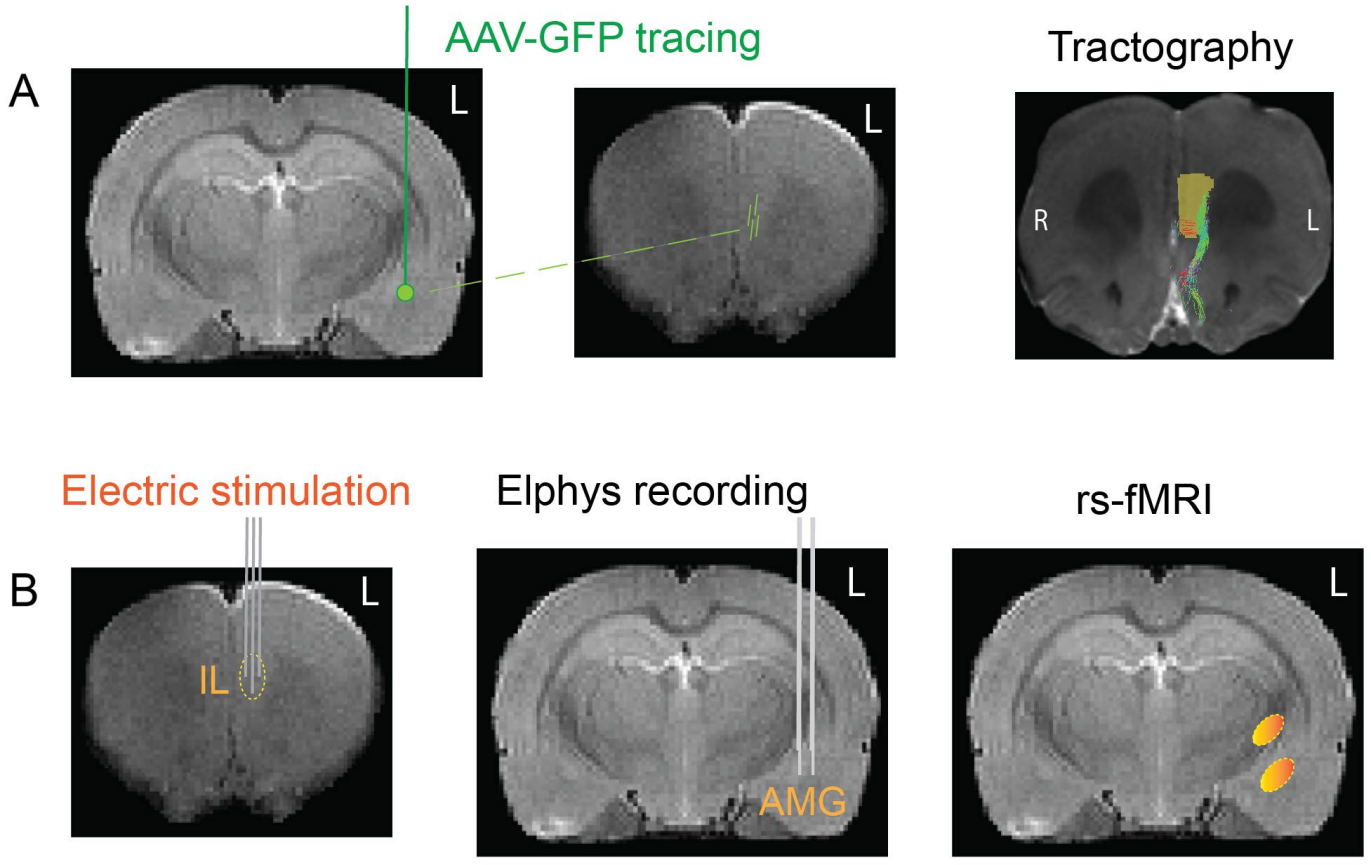
Schematic summary of the task protocol. **(A)** To map the orientations of the fiber tracts connecting the IL to the AMG, we injected adeno-associated viral vector with GFP into the left AMG and followed the spread of the virus to the left medial frontal cortex. In addition, we took high-resolution MR images from the frontal cortex of same rats and performed computer-aided tractography to reconstruct the course of the fibers that continue to the AMG. **(B)** To test the connection functionally, we stimulated the IL with brief electrical pulses using the new tetrahedral electrode and recorded local electrical field responses in the AMG with two parallel electrode bundles of three staggered wires. Finally, we selected the most effective vs. least effective angle of stimulation and stimulated the IL electrodes with the clinically relevant 130 Hz frequency and assessed the induced changes in resting-state MRI.

The approach of the present study is summarized in Figure 2. Based on these existing data, we first determined how the angle of the stimulation current vector in the IL modifies the local evoked responses in the AMG. Then, we chose the most effective stimulation orientation and assessed its ability to activate IL connected brain wide resting-state networks using a sustained subthreshold current and the clinically relevant 130 Hz frequency. To help interpretation of responses in functional connectivity, we stimulated only the left IL, which we did not expect to evoke measurable changes in the rat behavior. The data provide evidence for large variation in a distant monosynaptically connected target responses to a constant DBS current applied to IL while varying only the current vector orientation.

## 2. Materials and methods

### 2.1. Animals

In total, 15 Sprague-Dawley rats (350–400 g) were included in the experiments. We used only one sex to minimize the variation in the stereotactic electrode positioning. The rats were maintained under standard laboratory conditions (temperature 22°C ±1 °C, humidity 50%–60%, lights on 07:00–19:00) with food and water available *ad libitum*. All animal procedures were carried out in accordance with the EU Directive 2010/63 and approved by the Animal Experiment Board of Finland.

### 2.2. Surgeries

**Figure 3:**
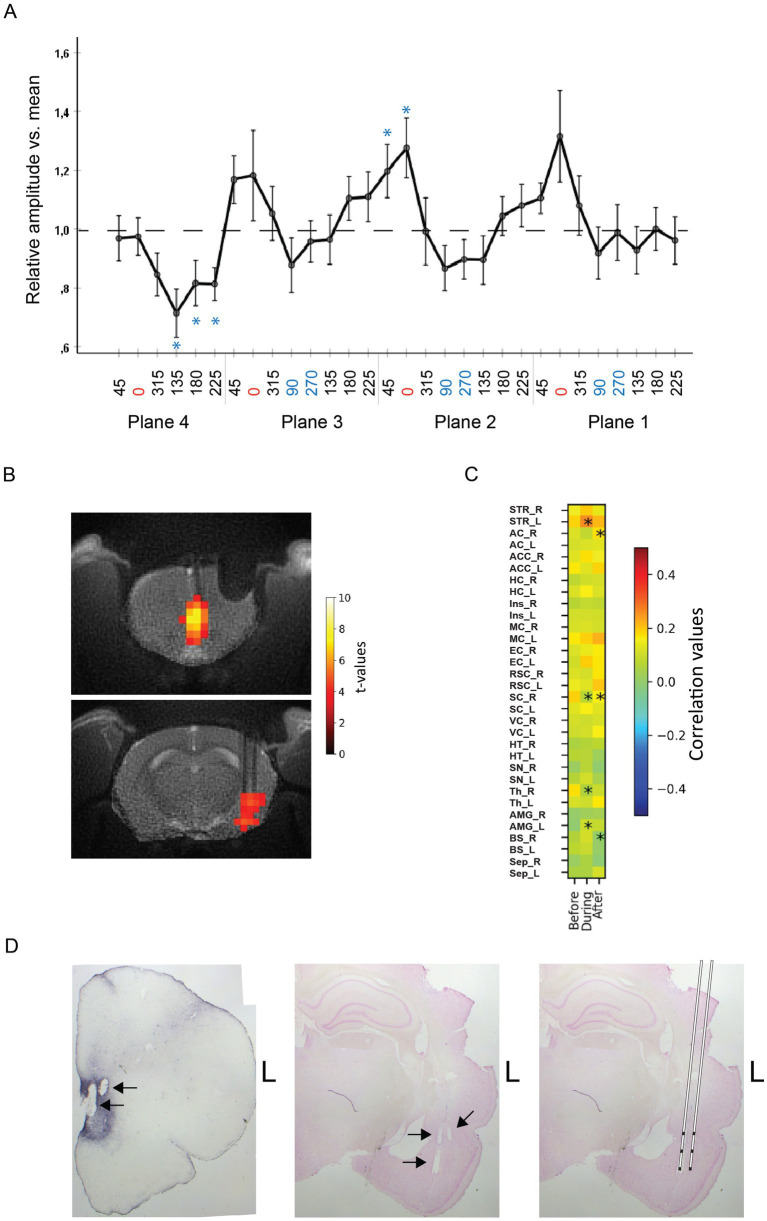
**(A)** Average evoked AMG responses to IL stimulation in 15 rats as a function of the stimulation current vector angle. The peak responses coincide with the most vertical orientations on planes 1–3, whereas no clear peak orientation can be seen on plane 4 that is tangential to the skull surface. The response amplitude is normalized to the mean amplitude for all stimulation angles in a given rat. The error bars indicate SEMs. *Significant difference from the group mean (one-sample t-test). **(B)** Example of MB-SWIFT activation maps of one rat in slices at the level of IL (top) and AMG (bottom) in response to suprathreshold 130 Hz IL stimulation at the most effective current vector angle. **(C)** Resting-state fMRI functional connectivity between IL and rest of the brain, during vs. before and after vs. during a 10 min of 130 Hz subthreshold stimulation at the most effective current vector angle vs. no stimulation. Asterisks indicate a significant change from the baseline. STR, striatum; AC, auditory cortex; ACC, anterior cingulate cortex; HC, hippocampus; Ins, insular cortex; MC, motor cortex; EC, entorhinal cortex; RSC, retrosplenial cortex; SC, somatosensory cortex; VC, visual cortex; HT, hypothalamus; SN, substantial nigra; Th, thalamus; AMG, amygdala. Note the increased connectivity between IL and AMG and between IL and AMG on the stimulation side (L) during the stimulation. **(D)** Histological verification of the stimulation electrodes in IL (left) and recording electrodes in AMG (middle). Reconstruction of the position of the two-shank, triple recording electrodes (right).

At the age of 5 months, the rats underwent electrode implantation in the stereotactic apparatus (David Kopf Instruments, Tujunga, CA, United States) under medetomidine (Dormitor, OrionPharma, Orion Corporation, Espoo, Finland) + ketamine (KetaminolVet, Intervet, Hoddesdon, UK) cocktail 0.02 mg/ml, s.c., with constant infusion at the speed of 1 ml/min). A custom-made stimulation probe consisting of four insulated (diameter 127/200 μm) tungsten wires (Bilaney, Hamburg, Germany) was targeted to the left IL at the following coordinates: AP (from bregma) +3.0 mm, ML +0.2 mm, DV (from dura) −4.6 mm (Figure 3D). In the same anesthesia, a 6-channel custom-made recording probe made of 50 μm insulated nichrome wire (California Fine Wire, Grover Beach, CA, United States) was implanted to the left AMG. The probe consisted of two bundles of three electrode wires with a tip separation of 600 μm. The lateral bundle was inserted into AP (from bregma) −3.2, ML +5.5, DV (from dura) −7.8 and aimed at the lateral amygdala, while the medial bundle was inserted to AP (from bregma) −3.2, ML +5.0, DV (from dura) –8.4 and targeted the central nucleus (Figure 3D). In addition, two cortical screw electrodes (brass, 1 mm diameter; Bergeon SA, La Chaux-de-Fonds, Switzerland) fixed on the parietal bone served as the indifferent and ground electrodes. The implant was fixed to the skull bone using self-curing resin (SelectaPlus; DeguDent GmbH, Hanau-Wolfgang, Germany).

Animals were given 0.9% NaCl injections (2 mL; s.c.) every hour during the surgery. Additionally, rats were given carprofen i.p. (10 mg/kg; Norocarp vet; Norbrook, Newry, Northern Ireland, United Kingdom) after the surgery and every 24 h for 2 consecutive days. After the implantation, rats were given 2 weeks to recover. Postoperatively all rats were singly housed to eliminate the implant damage by the cage neighbor. Cage enrichment was provided.

### 2.3. Orientation selective stimulation and local field potential recording

After the implantation and recovery period animals were handled and habituated for at least two days to recording conditions. During LFP recording, the rat was connected to the recording and stimulation cables and placed back to their home cage, but this time with elevated walls. LFPs were recorded only during waking immobility.

To enable free selection of the primary electric field direction during OSS, we designed a custom 4-channel probe where the electrode tips form an equilateral tetrahedron, with 300 μm separation between the edges (Figures 1A,B). The probe consisted of three shorter tungsten wires (Ø 150 μm with insulation) glued around a central 300 μm longer one. The central on was cut to length under stereomicroscope with a μm scale bar. The orientation of the electric field was controlled by inducing an electric dipole with a maximum of three electrodes at a time as described earlier (Lehto et al., 2018). For the orientation selective stimulation, the relative current amplitudes I_1,2,3_ of the three channels were chosen based on sinusoidal functions with phase offsets of 120° so that I_1 =_ I_0_ sin ф, I_2_ = I_0_ sin (ф + 120^o^) and I_3_ = I_0_ sin (ф − 120^o^), where I_0_ is the stimulation current amplitude and ф governs the stimulation angle. This induced an electric dipole field gradient such that the principal direction is defined by the phase step along the sinusoids. The current distribution between the three stimulation electrodes was chosen so that it created incremental steps of 45^o^ rotation on the plane connecting the three electrodes as follows: 0^o^, 45^o^, 90^o^, 135^o^, 180^o^, 225^o^, 270^o^, and 315^o^ (Figure 1C). To acquire orientation-selective DBS, 100 μs single bipolar square pulses were delivered at 0.1 Hz to each active channel of the stimulation probe at a variable current amplitude between 120 and 500 μA/channel, leading to a total current of ±500 μA (Figure 1D). To avoid adaptation to the stimulation, 2–3 min breaks were kept between each period of stimulation-recording (angle). We collected 20 evoked AMG responses for each of the 4 × 8 stimulation angles for averaging. The stimulation waveforms were computed using MATLAB (R2019b; Mathworks; Natick, MA, United States) and delivered through three stimulus isolators (model A365; WPI, Sarasota, FL, United States) connected to the stimulator (GRASS model S88; A-M System, Sequim, WA, United States).

Signals were normalized to amplification, and off-line analysis was conducted using custom-made scripts in MATLAB. To separate the local LFP in the AMG from more distant sources, we subtracted the voltage between two adjacent electrodes (Ch2–Ch1 and Ch3–Ch2) in a bundle that were vertically separated by 600 μm. The larger one of the local evoked responses was selected for analysis. We confined our analysis on the more laterally located electrode bundle that yielded more consistent responses. The voltage between the positive and negative peaks 5–30 ms after the stimulus onset was taken as response amplitude for each stimulation angle (Figure 1C). If the response was biphasic, we calculated the response amplitude as the difference between the peak and the trough, if monophasic, the difference between the peak/trough and the baseline. We also compared the power spectral density of LFPs during 1 s before and after the stimulation but found no evidence of after discharges with the applied stimulation strengths.

At the end of the experiment, positive DC current (30 μA for 5 s) was passed through the wire electrodes under the deep Equithesin anesthesia. The rat was transcardially perfused and the brain was prepared for histology. The electrode locations were confirmed in 35 μm coronal sections stained with cresyl violet.

### 2.4. Resting-state functional MRI during OSS

Functional MRI data were acquired in a high-field 9.4 T/31 cm bore magnet interfaced with an Agilent DirectDRIVE console (Palo Alto, CA, United States) using a custom-made (by Neos Biotec, Pamplona, Spain) surface transmit-receive radio frequency coil with a 22-mm inner diameter (ID). All imaging was performed with a 12-cm ID gradient coil set. First, high-resolution anatomical images were obtained using the fast spin-echo pulse sequence with the following parameters: TR 3 s, echo spacing 12 ms, effective TE 48 ms, number of echoes 8, matrix size 256 × 256, FOV 4.0 × 4.0 mm^2^, and 25 slices with 1 mm thickness. For functional measurements, we used the recently developed Multi-Band SWeep Imaging with Fourier Transformation (MB-SWIFT) fMRI that allows acquired fMRI responses comparable to those obtained with spin-echo (SE) echo-planar imaging (EPI) with minimal susceptibility artifacts from implanted metal wire electrodes (Lehto et al., 2017a,b). Functional MRI data were acquired with the following parameters: TR 0.97 ms, 2036 spokes per volume resulting in a temporal resolution of ~2 s, excitation/acquisition bandwidths 192/384 kHz, matrix size 64 × 64 × 64, FOV 4.0 × 4.0 × 4.0 cm^3^, and flip angle 5^o^. During MRI, the body temperature was maintained ~37°C with a warm water circulation system (Corio CD, Julabo, Seelbach, Germany). Small animal physiology monitoring equipment was used to follow the respiration rate and temperature of the anesthetized rats (Model 1025, Small Animal Instruments Inc., New York, NY, United States).

Out of 32 possible primary electric field directions, we chose the most effective and the least effective angles for the fMRI experiment based on the LFP recordings. To find the optimal stimulation current strength, we selected the most effective stimulation angle and applied 130 Hz stimulus trains (0.1 ms pulses, on for 18 s, off for 60 s, repeated three times), first at 200 μA current, then increasing the current in 100 μA steps up to 800 μA or until a local activation in the IL was visible. The protocol was the following. First, the 130 Hz stimulation was applied at the most effective field direction as described above to find the optimal current strength. A block measurement with the least effective field direction at the same current strength was performed to confirm orientation selectivity. Next, 10 min of resting-state fMRI (rsfMRI) was measured without any stimulation. Then the current strength was set 50 μA lower than the IL activation threshold and a 130 Hz continuous stimulation was applied for 10 min with fMRI recording. At the end, rsfMRI was taken again for 10 min without any stimulation.

The fMRI data were preprocessed using in-house written MATLAB code, Aedes software v217,^1^ ANTs,^2^ and Python 3.6.9.^3^ First, MB-SWIFT data were reconstructed using SWIFT package.^4^ Block stimulation datasets were analyzed with block analysis as implemented in Aedes to determine if the activation is present.

Resting-state datasets were motion corrected using ANTs and co-registered to the reference brain image. Signal from the electrode implant was regressed to improve data quality. Regions of interest were drawn on the reference brain, including IL, motor, somatosensory, anterior cingulate and insular cortex, striatum, hypothalamus, septum, thalamus, AMG, hippocampus, auditory, retrosplenial, entorhinal and visual cortex, substantial nigra, and brainstem. Correlation matrices between IL and the rest of the brain were acquired from the resting-state data before, during and after the continuous IL stimulation.

### 2.5. Tracing IL-AMG connection with viral vector injections

Additional 6 male Sprague–Dawley rats of the same age were given under general anesthesia stereotactic microinfusions of adeno-associated virus vector with the gene for green fluorescent protein (AAV-GFP; 250 or 500 nL/injection) to the central AMG at the following coordinates: AP (from bregma) −3.2, ML +5.0, DV (from dura) −7.3 for the tracing of fibers from AMG to prefrontal cortex. After 6 weeks, the rats were deeply anesthetized with Equithesin and transcardially perfused with ice-cold saline to rinse blood from the cerebral circulation and then with 4% paraformaldehyde solution. The brains were removed and immersed for 4 h in 4% paraformaldehyde solution. Then, the brains were placed in saline for at least 12 h to remove excess paraformaldehyde and underwent *ex vivo* diffusion MRI (see below). After *ex vivo* imaging, the brains were placed in 30% sucrose overnight. Next, the brains were left in an antifreeze solution at –20°C until cut into 35-μm coronal sections with a freezing slide microtome (SM2000R, Leica Biosystems, Buffalo Grove, IL, United States).

### 2.6. Tractography

In 4 out of 6 rats with AAV-GFP injections, *ex vivo* diffusion MRI data were acquired in a vertical 9.4 T/89 mm magnet (Oxford Instruments PLC, Abingdon, United Kingdom) interfaced with a DirectDrive console (Varian Inc., Palo Alto, CA, United States) using a quadrature volume RF-coil (Ø = 20 mm; Rapid Biomedical GmbH, Rimpar, Germany) as a transceiver. During the scans, the brains were immersed in perfluoropolyether (Solexis Galden®, Solvay, Houston, TX, United States) to avoid signal from outside the brain. We used a 3D SE-EPI sequence with the following parameters: TR/TE = 300/25 ms; number of segments = 8; number of averages = 2; FOV = 14.4 × 12.8 × 19.2 mm^3^, matrix size = 144 × 128 × 192, spatial resolution = 100 × 100 × 100 μm^3^; 3 b-values of 1,500, 2,500 and 4,500 s/mm^2^ were applied with 17, 30, and 35 directions, respectively, along with 3 non-diffusion weighted volumes (b = 0 s/mm^2^); δ = 4.9 ms, and Δ = 10.8 ms. Tractography was performed to assess the orientation of the fiber bundles connecting the IL/PL region and AMG. The response function for white matter was calculated using Dhollander’s algorithm (Dhollander et al., 2016) to perform multi-shell multi-tissue constrained spherical deconvolution (Jeurissen et al., 2014). Probabilistic tractography was performed using the fiber orientation distribution by seeding two million streamlines within a manually defined region of interest in IL. Then, from the resulted tractogram, we used spherical deconvolution informed filtering of tractograms (SIFT; Smith et al., 2013), to estimate the streamlines that are robustly supporting the data. The processing of the diffusion data and the generation of the tractograms were done using MRtrix3 (Tournier et al., 2019).

### 2.7. Statistics

The data were statistically analyzed by using IBM SPSS statistics 27 (IBM, Armonk, New York, NY, United States). To create a tuning curve for orientation selectivity, the AMG responses to all angles were first normalized by dividing them with the grand mean response of each rat. Orientation selectivity was tested with ANOVA for repeated measures with the orientation vector angle as the within-subject parameter. In three rats, the voltages were not comparable between the planes due to a temporary failure of the stimulator to deliver sufficient current. Those rats were excluded from the analysis. In the resting-state fMRI connectivity analysis, the correlation values before and during stimulation, and also during and after stimulation, were compared using paired *t*-test (FDR-corrected for multiple comparisons).

## 3. Results

### 3.1. Vertical stimulation field is the most effective, horizontal the least effective

All rats showed local AMG responses to at least some of the 32 different stimulation orientations. As seen in Figure 1C from one example case, varying the stimulation angle on the horizontal Plane 4 had little influence on the local AMG response, whereas the amplitude variation was large in all three vertical planes (30^o^ deviation from the vertical axis to be precise). In contrast, the field orientation had little impact on the response latency. To test the consistency of how the AMG response depended on the current field orientation in IL, we normalized the response of all 15 rats in the study and ran ANOVA for repeated measures with the stimulation field angle as the within-subject factor. The ANOVA proved significant (*F*_1,29_ = 2.5, *p* = 0.04, Greenhouse-Gasser correction), speaking for such a consistency at the group level. As can be seen in Figure 3A, the peak responses coincide with 0^o^ angle in Planes 1–3, i.e., the angle that is closest to the dorso-ventral direction. However, such a peak is missing on the horizontal Plane 4. Further analyses showed that the dorsal to ventral 0^o^ angle differs marginally from the two horizontal angles (90^o^ and 270^o^; *F*_1,14_ = 4.5, *p* = 0.05) but not from the opposite ventral to dorsal (180^o^) direction (*F*_1,14_ = 2.5, *p* = 0.13). These data speak for the idea that more vertically oriented fields evoke the largest local responses in AMG.

However, when considering individual rats, there was still quite a lot of variation. Table 1 shows the most and least effective stimulation field angles for each rat. For 8 rats out of 15, a vertical field was the most effective, while only for 6 rats a horizontal field was the least effective.

**Table 1:** The most and least effective current field vector angles in the infralimbic cortex to evoke amygdala responses in each rat.

	Most effective		Least effective	
Rat ID	Plane	Angle	Plane	Angle
2	1	45	3	135
7	1	0	1	225
8	1	0	4	0
10	2	0	2	90
18	4	0	1	315
19	3	180	2	315
20	2	180	3	0
45	3	0	4	135
46	1	135	3	0
47	3	0	1	270
48	1	0	4	135
49	3	225	4	180

### 3.2. Orientation selectivity of AMG responses concur with fiber orientation within IL

**Figure 4:**
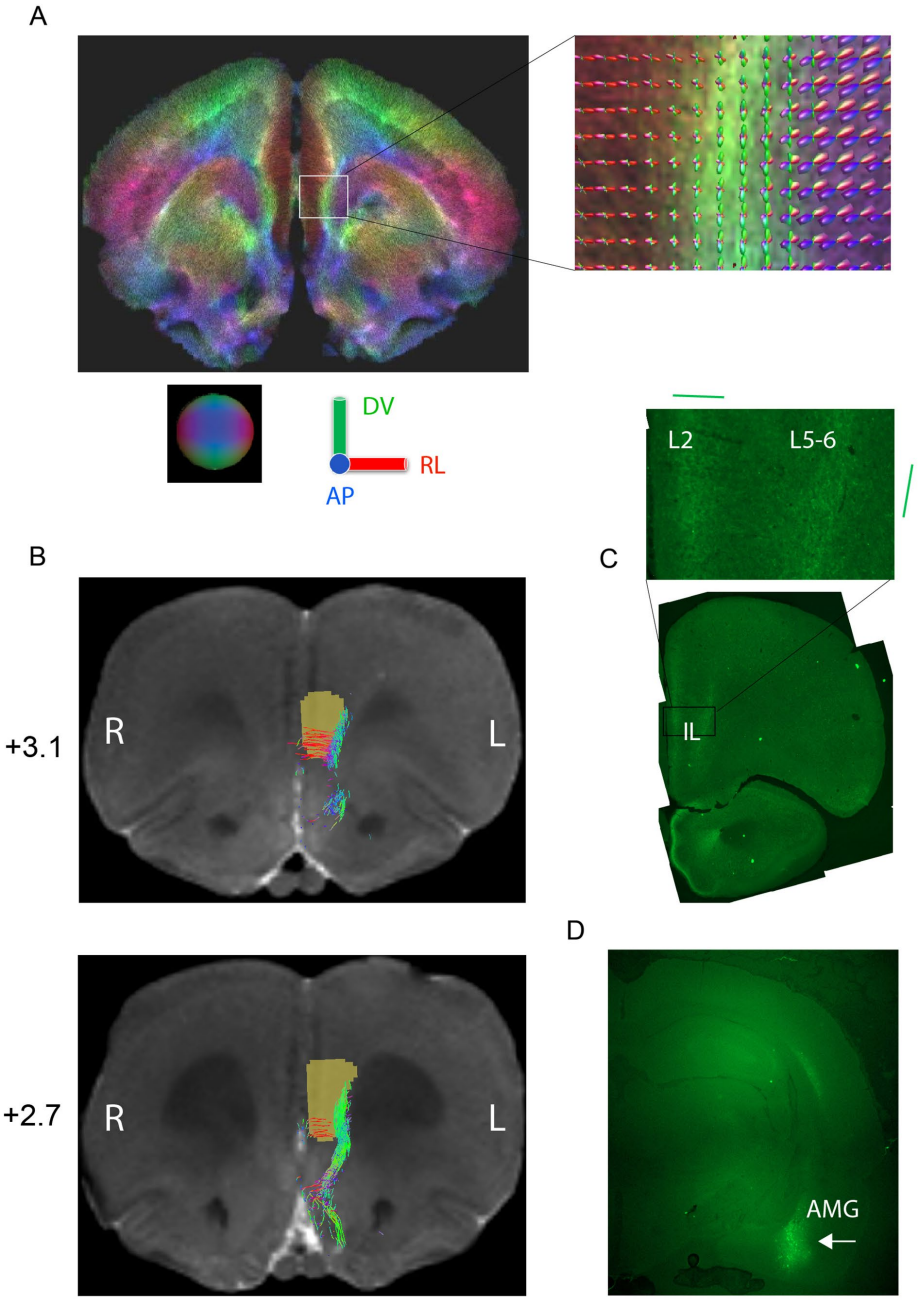
Tracing of connections between infralimbic cortex (IL) and amygdala (AMG). **(A)** Whole brain tractography of a rat *ex vivo* brain at the level of IL. The color code (as demonstrated by the sphere and colored axes) indicates different principal directions of fibers as follows: red = medial–lateral, green = dorsal–ventral, blue = anterior–posterior. The inset shows fiber orientation distributions in each voxel. **(B)** Probabilistic tractography of fibers connecting IL with AMG shown on two 100-μm coronal MRI slides (distance from bregma indicated). The brown region-of-interest was used as a seed in the propagation of the tracts. **(C)** Example of histological tracing of AMG to IL connections by an AAV-GFP viral vector. Inset: a close-up of the GFP-labeled terminals. The fibers in layer 2 have mainly a medio-lateral orientation while those in layers V-VI have a dorso-ventral orientation, both in agreement with the tractography **(D)** injection site in AMG (white arrow).

To better understand the relationship between the stimulation field direction in IL and AMG responses, we studied more closely the orientation of fiber bundles connecting IL with AMG (Vertes, 2004). First, we injected the green fluorescent protein (GFP) gene into AMG unilaterally with the help of an AAV vector. After 6 weeks from the injection, we perfused the rats and found GFP labeled axon terminals concentrate on two bands in IL, layer 2 near the brain midline and layers 5–6 (Figures 4C,D). The main orientation of these labeled terminals was medio-lateral in layer 2 but dorso-ventral in layers 5–6. Second, we approached the question also by doing diffusion MRI on the same rats (Figure 4A) followed by tractography with IL as the defined origin and AMG as defined termination area. The results were highly consistent with the tracing study: in IL, layers 1–3 contain AMG reaching fibers running in the medio-lateral direction, whereas the fibers in layers 5–6 have a dorso-ventral course in the posterior IL, but also include some anterior–posterior orientation in rostral parts of IL (Figure 4B). Since the estimated probe center in the ML direction was 0.49 ± 0.03 from the midline surface in our rats, this means that the probes were located in the more vertically oriented lateral band of AMG axon terminals. This may well explain why on average the vertically oriented fields yielded a stronger AMG response than more horizontally aligned. On the other hand, the presence of fibers in all three main 3D axes in a small tissue volume with variation in the exact probe location was compatible with the individual variability in the most and least effective stimulation angles.

### 3.3. Orientation selective fMRI responses in AMG after IL stimulation

After determining the most effective stimulation field orientation in each rat using electrophysiology, we went on to assess whether the same stimulation field orientation would be effective in a more clinically relevant setting. We chose 7 rats with a clear orientation selective response profile and administered the clinically relevant 130 Hz stimulation in three 18 s trains at incremental current strengths until a significant activation in the brain was detected (Figure 3B). Next, we reduced the current by 50 μA to be more suitable for a sustained stimulation. Finally, we delivered sustained stimulation at 130 Hz for 10 min and tested the effect of the orientation optimized IL stimulation on brain-wide resting-state connectivity. We compared a 10 min baseline period without any stimulation with 130 Hz continuous stimulation for 10 min, and finally its possible after-effects by another 10 min period without stimulation. The effects on resting-state connectivity with IL are summarized in the connectivity matrix in Figure 3C. The connectivity matrix included brain regions with monosynaptic connections with IL (Vertes, 2004; Wood et al., 2019). The unilateral (left) stimulation resulted in significantly increased connectivity between left IL and left AMG, as well as between left IL and left striatum (including nucleus accumbens), regions that are monosynaptically connected with IL.

## 4. Discussion

The present work set out to investigate whether orientation-selective DBS could provide a means to optimize the electric current so that only a select neural tract is activated in a brain region with intercrossing fiber bundles. To this end, we designed a simple four-wire stimulation probe where the tips formed a tetrahedron. We focused on IL of the rat, an analogous brain region to human sgACC, which has been identified as a promising but anatomically challenging DBS target in the treatment of drug-resistant depression. As is the case with the human sgACC, the rat IL is an intersection with highly variable axonal fiber orientation. The main finding was that upon IL stimulation a given current strength yielded variable local evoked responses in AMG depending on the primary field orientation.

At the group level, the most vertical field orientations yielded the highest AMG responses, whereas the horizontal orientations evoked on average the smallest responses. It is noteworthy, however, that among the horizontal orientations the highest AMG response was obtained with 45^o^, which is closest to the most effective horizontal orientation to evoke fMRI activation in AMG in our earlier work with a planar probe (note the probe was on the right side, here on the left; Lehto et al., 2018). Based on the probe location, tractography, and neural tracing, the near to vertical stimulation field orientation was aligned with the main axonal bundle connecting IL with AMG (Figure 4A). This is fully in line with a recent modeling study showing that an electrical dipole along the axon results in the strongest activations while the smallest one is evoked by a dipole at a right angle to the axon, and that evoked response depends on the dipole angle following a sigmoid curve (Slopsema et al., 2018). It is worth noticing that even when the stimulation electrode is located in the gray matter, as in our case, the electric field depolarizes not only the round neuronal somata but also their axons, which tend to be organized in parallel bundles even in within the gray matter (Figure 4A; McIntyre et al., 2004). Nevertheless, when considering the most effective stimulation orientation for each individual rat, this was opposite to the best group orientation in two and an intermediate angle in three out of 12 rats. Also in this regard, the situation resembled the clinical setting. Although individual differences in neuroanatomy play a much smaller role in inbred rats than in human patients, small differences in the exact stereotactic coordinates and the angle of the probe during the implantation account for the individual variability. The power of the orientation selective stimulation was the ability to balance the individual differences by adjusting the orientation of the current dipole.

Notably, for practical reasons, we only used 3 out of 4 probe tips when delivering the current. Thus, the stimulation field directions only covered each of the tetrahedron faces in eight 45^o^ steps. If we had included stimulation with all 4 tips active at the same time, we could have set the current field at any direction in the 3D space. The most notable direction that was not covered was the exact vertical direction. Theoretically, this could be achieved easily by shortening all short electrode wires and pairing it with the longest one at either polarity. Unfortunately, this was not possible on the run with our setup. Therefore, our selection for the most and least effective current field orientation was not fully optimized. Here, we were also limited by the time it took to test all 4 × 8 orientation in the same session. In contrast, in the clinical setting the stimulation current and the field orientation could be first set grossly at the right level and later fine-tuned in several sessions. Furthermore, the probe implantation is often done in awake patients with the possibility to test the immediate response by intraoperative stimulation.

To simulate the clinical situation, we first verified the orientation selectivity and searched for the optimal current at 130 Hz stimulation that in fMRI activated the target region of the stimulated pathways (Lehto et al., 2018). Then, we reduced the current and delivered a clinically widely used 130 Hz sustained stimulation that also has a documented antidepressant effect in rats (Hamani et al., 2010; Jiménez-Sánchez et al., 2016). Although this stimulation frequency did not result in any direct evoked activation in fMRI at a subthreshold current, the analysis of resting-state functional connectivity with the stimulation site (IL) resulted in significant changes during 10 min of sustained stimulation. Notable, significantly increased connectivity with IL was found in AMG and striatum (also including nucleus accumbens), both of which are known to be monosynaptically connected with IL (Johansen-Berg et al., 2008; Wood et al., 2019). We chose unilateral IL stimulation with the 130 Hz as it allowed assessment of the lateralization of the effect on IL seed based resting-state network. The downside of the unilateral stimulation was that it was not expected to result in significant behavioral changes that could be interpreted as antidepressive. As this was also noticed in our pilot studies, we did not include a behavioral test battery to this protocol. It remains to be seen in further studies whether earlier described antidepressant effect of bilateral IL stimulation are orientation dependent to the same extent as the currently applied unilateral stimulation.

**Figure 5:**
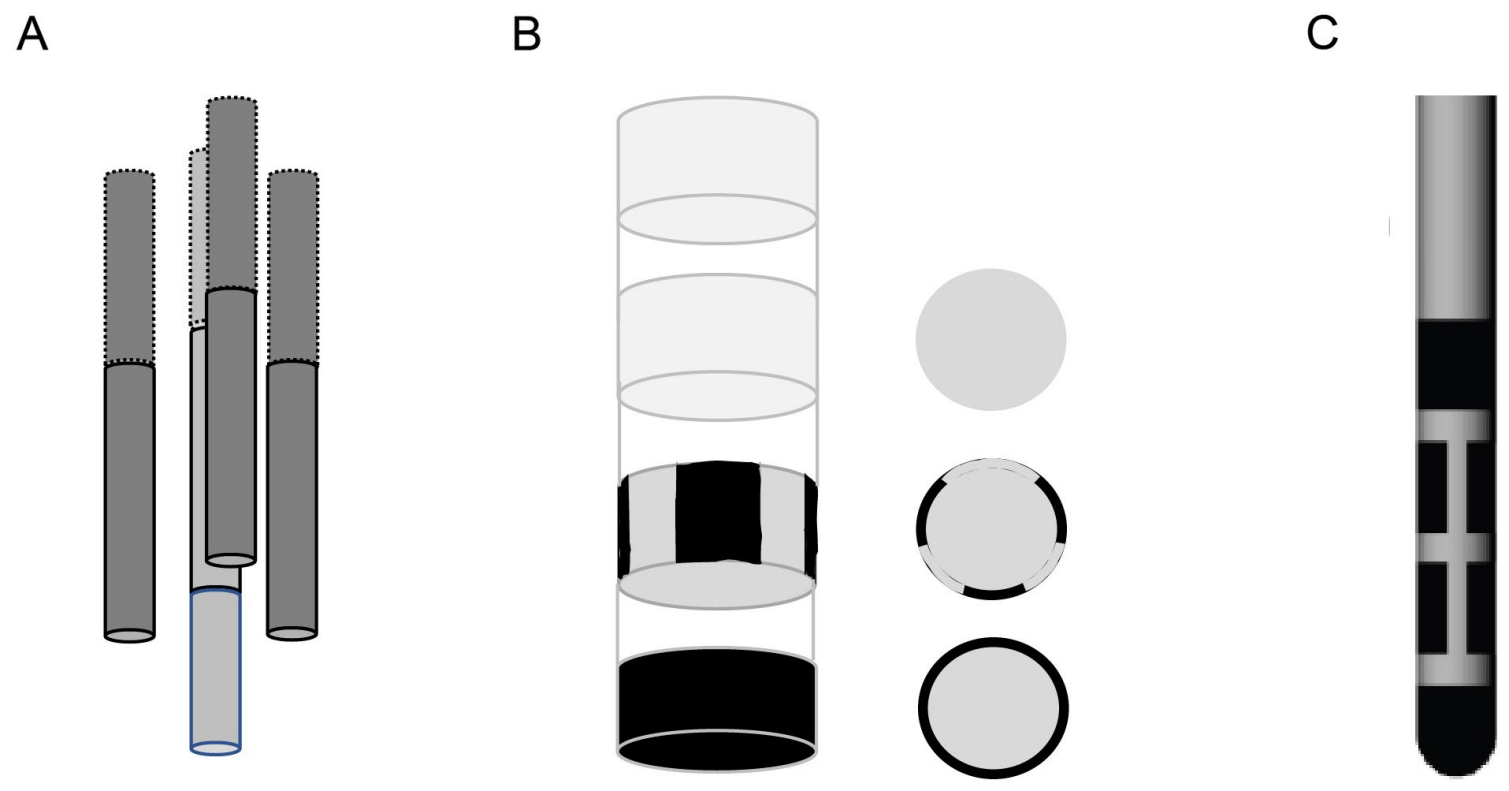
**(A)** Schematic drawing of the tetrahedral 4-electrode probe used in this study. **(B)** Schematic drawing of a directional commercial probe aimed for human use at the same viewing angle as the tetrahedral probe. **(C)** Side view of the typical commercial directional DBS probe.

Finally, the principle of 3D-OSS as presented here is applicable to the present clinical directional probes that basically consist of two tetrahedral assemblies of electrodes in mirror images (Figure 5). The only concern is the probable impedance difference between the electrodes, as the tip and base electrodes have a larger surface area than the segmented central electrodes. However, this can be adjusted in the calculations of stimulus voltages to obtain matched current intensities.

Collectively, these findings suggest that a simple probe design with only four active electrodes in the proper spatial arrangements create current dipoles that can be freely adjusted to any 3D angle in the volume near the probe. The ability for orientation selectivity will greatly increase the possibilities to optimize deep brain stimulation in brain regions with many intercrossing fiber paths to the needs of an individual patient. This ability mainly helps avoiding stimulation of pathways with undesirable (usually motor) effects. However, having better spatial selectivity of the stimulation does not change the non-selective activation of cells within the volume of cells that are fired by the stimulation.

## Data availability statement

The raw data supporting the conclusions of this article will be made available by the authors, without undue reservation.

## Ethics statement

The animal study was reviewed and approved by Animal Experiment Board of Finland.

## Author contributions

IG, HL, and ON: investigation and formal analysis. EP: formal analysis. LL: methodology. KG: software. SMa, SMi, OG: conceptualization, writing—review and editing, and funding acquisition. AS: methodology and writing—review and editing. HT: formal analysis, writing original draft, and project administration. All authors contributed to the article and approved the submitted version.

## Funding

This project was supported by the National Institutes of Health (NIH) grant U01-NS103569-01, the Center for Magnetic Resonance Research NIH core grant P41 EB027061, and Jane and Aatos Erkko Foundation (Finland).

## Conflict of interest

The authors declare that the research was conducted in the absence of any commercial or financial relationships that could be construed as a potential conflict of interest.

## Publisher’s note

All claims expressed in this article are solely those of the authors and do not necessarily represent those of their affiliated organizations, or those of the publisher, the editors and the reviewers. Any product that may be evaluated in this article, or claim that may be made by its manufacturer, is not guaranteed or endorsed by the publisher.

## Footnotes

^1^ http://aedes.uef.fi

^2^ http://stnava.Github.io/ANTs/

^3^ https://www.python.org/

^4^ https://www.cmrr.umn.edu/swift/index.php
